# Post-Mortem Examination of a Case of Chronic Hydrocephalus

**Published:** 1831-02

**Authors:** Marshall Hall


### TAPPING IN HYDROCEPHALUS.

### *Post-mortem Examination of a Case of Chronic Hydrocephalus.* By Marshall Hall, M.D. &c.

There was no very obvious appearance externally, to lead to the supposition that one side of the head, or one ventricle, was more distended than the other.

On a careful examination of the internal parts, however, it was found that the effusion chiefly occupied the right ventricle, which was at least three times more distended than the left.

The effect of this circumstance was, that the falx, and of course the longitudinal sinus, by no means occupied the mesial plane.

If, therefore, the operation of paracentesis capitis had been performed, or if a point had been fixed upon for the puncture, on the upper part of the head, the trocar, in being inserted on the left of the mesial plane, might have penetrated into the substance of the brain, or even into the longitudinal sinus itself.

It is important to remember that, if a line be drawn *touching* the upper parts of the parietal and frontal bones, *on either side,* it may exactly cover the longitudinal sinus. In order to avoid this sinus, therefore, we must leave the line just spoken of, and choose a spot still further removed from the mesial plane, and within the angle formed by the anterior part of the parietal and posterior edge of the frontal bones.

On a still more careful external examination of the head, I think it not unlikely that one side may be found distinctly more protuberant than the other, its bones less ossified, less approximated together, and less *formed.* Such indications of greater distension of one ventricle than of the other, must be important in fixing our choice of the *side* of the head for the operation, whatever the *point* selected may be, which I do not presume to determine.

This case appears to me additionally interesting at the present moment, as, from the recent success with which Dr. Conquest has tapped the head in cases of hydrocephalus,* the operation will probably be more frequently resorted to in future.

* London Med. and Phys. Journal, May and December, 1830.

